# Ten simple rules for using public biological data for your research

**DOI:** 10.1371/journal.pcbi.1010749

**Published:** 2023-01-05

**Authors:** Vishal H. Oza, Jordan H. Whitlock, Elizabeth J. Wilk, Angelina Uno-Antonison, Brandon Wilk, Manavalan Gajapathy, Timothy C. Howton, Austyn Trull, Lara Ianov, Elizabeth A. Worthey, Brittany N. Lasseigne

**Affiliations:** 1 Department of Cell, Developmental and Integrative Biology, Heersink School of Medicine, The University of Alabama at Birmingham, Birmingham, Alabama, United States of America; 2 Center for Computational Genomics and Data Sciences, Heersink School of Medicine, The University of Alabama at Birmingham, Birmingham, Alabama, United States of America; 3 Department of Pediatrics, Heersink School of Medicine, The University of Alabama at Birmingham, Birmingham, Alabama, United States of America; 4 Department of Pathology, Heersink School of Medicine, The University of Alabama at Birmingham, Birmingham, Alabama, United States of America; 5 Civitan International Research Center, Heersink School of Medicine, The University of Alabama at Birmingham, Birmingham, Alabama, United States of America; Dassault Systemes BIOVIA, UNITED STATES

## Abstract

With an increasing amount of biological data available publicly, there is a need for a guide on how to successfully download and use this data. The 10 simple rules for using public biological data are: (1) use public data purposefully in your research; (2) evaluate data for your use case; (3) check data reuse requirements and embargoes; (4) be aware of ethics for data reuse; (5) plan for data storage and compute requirements; (6) know what you are downloading; (7) download programmatically and verify integrity; (8) properly cite data; (9) make reprocessed data and models Findable, Accessible, Interoperable, and Reusable (FAIR) and share; and (10) make pipelines and code FAIR and share. These rules are intended as a guide for researchers wanting to make use of available data and to increase data reuse and reproducibility.

This is a *PLOS Computational Biology* Methods paper.

## Introduction

**Fig 1:**
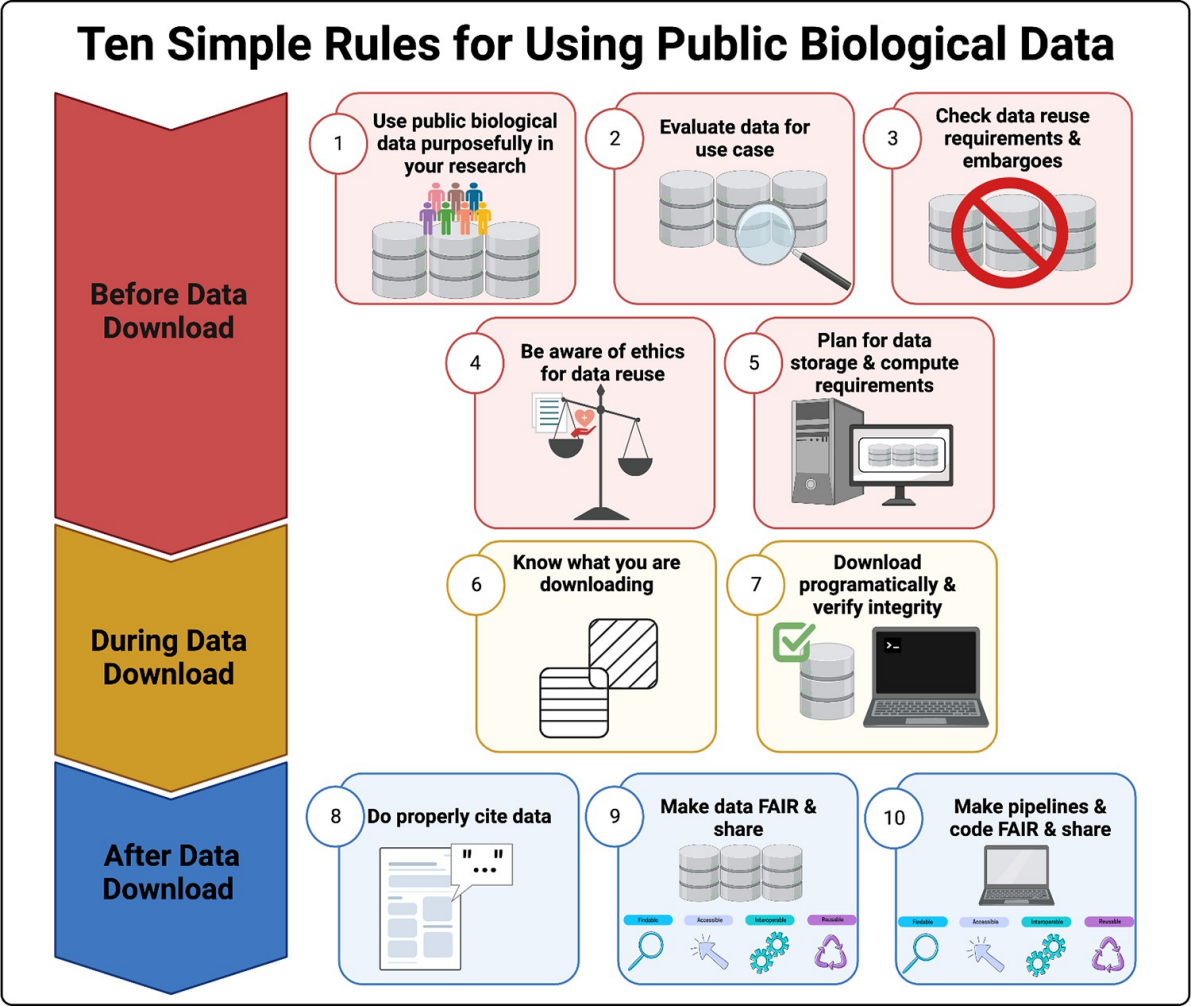
These 10 simple rules for using public data span checklist items for pre, during, and post data download.

In recent years, with the advent of high-throughput sequencing technologies, advances in microscopy, and the growth of single-cell technologies, biology is set to overtake other data-heavy disciplines such as astronomy in terms of data storage needs [1]. There has been a dramatic increase in the number and size of data sets deposited by individual labs on data storage servers like the Gene Expression Omnibus (GEO) and dbGAP [2,3] and made available by large consortium efforts such as The Cancer Genome Atlas (TCGA) [4], the Genotype-Tissue Expression (GTEx) project [5], Bgee [6], Human Cell Atlas [7], and ENCODE [8,9]. These resources provide rich and diverse data to the biology research community. For example, TCGA is made up of clinical information (e.g., smoking status), molecular analyte data (e.g., sample portion weight), and molecular characterization data (e.g., imaging data, omics data). The additional commitment by funders, publishers, and individual scientists to make data sets (especially those funded by taxpayers and donors) publicly available has rapidly increased opportunities for data reuse by the broader scientific community. From January 2023, NIH will now require researchers to share data generated with NIH funds under the new Data Management and Sharing Policy [10] further leading to an increase in the availability of public data. With this growing data deluge, it seemed timely to provide guidelines on why, when, and how investigators can incorporate these valuable resources into their research programs as biological data reuse is good for science, cost efficient, and is the right thing to do in order to extract the greatest societal impact from the samples and funding that patients, donors, and taxpayers generously provide. Here, we draw upon our collective experience and expertise as molecular biologists, computational biologists, bioinformaticians, data scientists, and software developers to discuss Ten Simple Rules for using public data with the intention that it will serve as a useful guide (Fig 1). While this article focuses on computational biology and bioinformatics, the principles outlined here generally apply to other domains as well.

### Rule 1: Use public biological data purposefully in your research

Reasons for reusing public biological research data include cost-effectiveness and efficiency, access to data sets that would be difficult or impossible to regenerate, an increased sense of community, greater transparency and clarity of research, ability to retest and validate a shared data set, support for recognition of data ownership, and an increase in citations [11]. Critically, public biological data reuse can also promote workforce diversity and research inclusivity worldwide [12–14]. Use of public biological data provides individuals with a unique opportunity to leverage existing data sets in combination with other public data or their own. This allows for additional contexts to be explored within your own research. Comparing your research findings with a public data set analyzed in the same way can give nuance or clarity to results, especially in either further confirming what was observed (i.e., validation) or producing different results that help you to re-evaluate your approach or its applicability across cohorts. For example, you can validate current findings in public data, expand the number of samples, age groups, or other parameters of your current analysis, or explore molecular changes in a different system. Assessing public data can also aid in placing your research into the context of, and in perspective to, current research with public data therefore allowing comparisons to other work in the field.

Incorporating public biological data in your research project can also allow you to ask novel questions that the data generator may not have originally foreseen, further moving your own research and the field in new directions. Outside of expanding your research through exploration of new contexts, public data sets can also be used to generate hypotheses and/or guide future research by refining hypotheses for preliminary analyses. However, be mindful of data being appropriate for answering your hypotheses as outlined in Rule 2. With computational biology becoming ubiquitous in life science research, you can also use public data to drive novel method development and build modeling frameworks for systems biology. In fact, there have been efforts to standardize data sets for ease of use in such analysis [15]. In addition to improving your analysis, using public data also provides an opportunity to practice reproducible and interoperable research and further develop professional data science skills [11]. However, it is important to avoid the “sins of methodological research” (i.e., selective reporting, lack of replication studies, poor design of comparison studies, publication bias) [16] and therefore we suggest ensuring your research team has the proper methodological expertise to properly use and interpret public biological data sets.

In short, novel studies do not always require new data, and research including data reuse not only offers all of the benefits mentioned here, but can support community building and greater impact; as shown by Milham and colleagues [17] who found that neuroimaging research making use of public data was cited at the same or higher frequency in higher impact journals as compared to research using only self-generated data (this is expanded upon more in Rule 8). By using public biological data with care, your research can benefit from these benefits.

### Rule 2: Evaluate data carefully for your use case

Before we get down to the business of downloading the data, we need to make sure we access appropriate data. As scientists, we are interested in finding signals and/or patterns in our data. However, patterns in a data set can arise from many different variables. Therefore, it is essential to carefully evaluate the available data for your particular use case; otherwise, the results obtained could be wrong and/or misleading. It is critical to examine the metadata (the data describing the data set) before downloading the actual data. Some important metadata variables to consider (but by no means exhaustive) relate to the (1) nature of the sample (e.g., the specific animal model or cell line(s), genotype, age, sex); (2) sample collection data (e.g., the type of sample collected, time points of collection); (3) platforms used (e.g., the sequencing or imaging platform); and (4) data quality metrics (e.g., sample size, groups of comparison). Not considering these variables can dramatically influence downstream analysis and interpretation and should therefore be considered before choosing the appropriate data set to use. For more details, see Rules 4 and 5 in “Ten simple rules for providing effective bioinformatics research support” [18].

Once the data set is deemed appropriate for your analytical goals, it is key to check for the confounding effects of metadata variables that might impact the statistical model and cause you to reach wrong conclusions. This includes review of data distributions and sampling as well as performing exploratory analysis and visualizations such as principal component analysis (PCA) and correlation analysis. “Ten simple rules for initial data analysis”[19] provides excellent pointers for performing initial data screening including how to craft a reproducible plan and pitfalls to avoid.

### Rule 3: Be aware of, and adhere to, data reuse requirements, embargoes, etc.

Researchers looking to reuse controlled-access data (elaborated more in Rule 4) assume the responsibility (along with their legal and financial administrative offices) to protect the rights and welfare of the original participants. As such, data repositories with controlled-access data sets develop Data Access Request (DAR) processes to appropriately restrict access. DAR processes usually include agreeing to terms of access for the requested data, a Data Use Certification Agreement (DUA) between the requester’s institution and the repository, a description of the intended use, and an acknowledgment agreement. If a data set is available in a data portal (e.g., GEO [2], SRA [20]) that does not require controlled access, it is considered open access and can be freely downloaded through the portal. Even under these circumstances, the original data generators may still request acknowledgment upon use (a practice that should be followed whether or not the generators have requested it). A third possibility for acquiring controlled-access data for reuse is to contact the corresponding author of the paper directly to request data. However, this method can prove difficult for many reasons related to the author’s availability to fulfill the request (e.g., protected time to fulfill the request, ability to transfer the data, willingness to comply). Therefore, independent of the method of requesting access to restricted data, it is important to be patient, but persistent. Recently, many journals have begun implementing data availability manuscript sections that outline the portal where the data is stored and can be accessed.

Regardless of how the data is acquired, it is important to be aware of the legal, regulatory, and security obligations associated with its use. For example, data licenses might be in place to protect the original data generator’s rights by permitting secondary parties to reuse the data according to specific restrictions. Because countries have different regulations for data reuse [21], data licenses may clarify the uncertainty of requirements for data reusers. It is important to understand by which license or waiver the data to be reused is governed. In an effort to promote open access to data, many journals and data repositories operate under Creative Commons (CC BY 4.0) licenses that allow the freedom to share and adapt data as long as the original data generators are acknowledged for their contribution. For example, PLOS ONE stipulates that if the data associated with a published article in their journal is deposited in a repository with a licensing agreement, the agreement cannot be more restrictive than CC BY [22]. Researchers should identify which, if any, data license is governing the data they wish to reuse and respect any limitations associated with it.

Some data generators place an embargo on the data they generate in order to ensure they have time to publish initial findings. The data set may be submitted to a public repository but unavailable for download or publication for a certain length of time or until a specified date. Additionally, with the increased prevalence of pre-printing articles, data generators may wish to withhold their data sets until their article is published in a peer-reviewed journal. Being aware of data access and publication restrictions associated with the data set of interest and understanding who sets data restrictions can be complicated (e.g., funders, consortia, journals, individual labs), but convention, or in some cases the legal requirement, is to follow the stated restrictions.

### Rule 4: Be aware of ethical considerations like confidentiality and protected health information

The privacy and ethics of data sharing are critical and it is the responsibility of researchers to protect and observe [23]. With regards to data reuse, you must verify that the data you are planning to reuse was ethically collected or generated, was collected for a purpose in alignment with additional applications, and ensure that the study does not use the data irresponsibly or immorally. It is our duty as scientists and citizens to move science forward while protecting sensitive data and presenting studies fairly. Secondary analysis of public data requires an Institutional Review Board (IRB) exemption confirming it does not fall within the regulatory definition of human subject research (see federal regulations on human subjects research protections (45 CFR 46.101(2)(b))) and a full review otherwise (e.g., software as a medical device where you are using AI or machine learning for diagnosis). Special considerations need to be made for data reuse:

- Ethics by data type: The ethics of sharing and using data depend heavily on the data type. For example, cell lines, animal models, and microbial data are typically very low risk for privacy or ethical issues; that is not to say, however, that they have no risk (e.g., sharing pathogenic sequence data or alluding to locations for endangered species that could increase the risk of poaching) [24]. Ethical concerns are most common when working with human data.
- HIPAA, PHI, and patient de-identification: The Health Insurance Portability and Accountability Act (HIPAA) of 1996 Privacy Rule is a federal law regarding protected health information (PHI) for individuals and their access to their own health information, as well as the specific permissible use and disclosure of PHI with other organizations [25]. Any purposeful or accidental disclosure of PHI as a HIPAA violation can lead to hefty fines. Health data used in research needs to be de-identified in a manner that makes re-identification highly unlikely unless that is the willful goal of the project. Genomics data is particularly susceptible to re-identification due to the uniquely identifying nature of the data itself; particularly when coupled with geographic collection metadata such as collection site. Well-constructed data usage agreements and controlled-access repositories can mitigate re-identification risks (see Rule 3).
- Consent: Consent is an agreement between healthcare groups and participants that adheres to The Privacy Rule and HIPAA authorizations. For example, the GTEx Live Donor Informed Consent Template (BBRB-PM-0018 [26]) asserts exclusion of access to participant PHI that the generated data will be saved for many years, that it will be available to scientists around the world, and that data may be used broadly for medical research. It is important to ensure that data reuse does not violate any initially obtained consent.
- Ethics specific to how/where data were obtained: Data may be obtained directly from an individual lab, or queried from a private or public repository. Publicly available data that can be downloaded by anyone tends to be at lowest risk for violating privacy and are the easiest to access (i.e., TCGA [4] and GTEx [5] gene expression data). However, when data is received from another investigator through direct sharing or from a controlled-access repository, it becomes your obligation to secure that data and not further share with unauthorized individuals [24].
- Ethical design for data reuse: When reusing data, ensure fairness and equality with your representation of the data, including but not limited to ancestry or sex. For example, genetic study participation has been disproportionately overrepresented by European descendants, where one study found that as of 2018, individuals in GWAS catalogs consisted of 78% European, 10% Asian, 2% African, 1% Hispanic, and <1% for all other races [27]. In GTEx as of 2022, 84.6% donors were of white origin, 12.9% African American origin, 1.3% Asian origin, 1.1% unknown and no statistic for Hispanic/Latino origin [5]. Additionally, sex differences impact every area of health and have been largely disregarded in study design and have also been heavily unbalanced, especially in pharmaceutical trials where women were previously excluded entirely due to potential pregnancies during trials [28]. Since then, women are now included in study designs, though sex specificity has not been accounted for leading to vastly more adverse drug reactions in women due to inappropriate drug and dose recommendations [29]. Careful consideration of these factors will lead to more rigorous and accurate results and avoid perpetuating these issues in research.

### Rule 5: Plan for needed data storage and compute requirements

Ask yourself, is my data “genomical” in size [30]? It is if you’re working in genomics, but regardless of your data, the amount of data being integrated in research is growing dramatically and the cost for storage and computation is at a rate not seen previously. Knowledge is power, and in this case, it is knowledge of the resources that will be needed *before* you need it that will benefit your work most. Data storage and computing hardware requirements should be determined and documented prior to downloading any data sets. This avoids potentially time consuming and expensive surprises by: (1) identifying gaps in the current infrastructure; and (2) allowing expert support staff an opportunity to investigate the viable solutions, where possible, in a timely manner. While needs vary by the individual situation and domain, here, we discuss various criteria to determine your needs. Data size, type, level of access, and security are the major considerations for where data needs to be stored. All invested parties who will retrieve, process, and consume the data need to be identified and involved in answering the following questions:

Data size:

- What is the estimated size of data to be retrieved?
- How many individual files are included?
- How much data are expected to be produced during processing?
- Do you need backups of the original or processed data?

Data type:

- Are the data types large or small?
- Are the file types binary or textual?
- Are the files compressed?

Access requirements:

- Who needs (or does not need) access to the data?
- What is the level of access required?
- How often will access be needed?
- How often will reanalysis be necessary?
- Do any of the users require (or prefer) a data sharing platform with a graphic interface for ease of access?
- Are there any institutional policies or approvals to be considered prior to granting access?
- Do external collaborators need to be provided access to the institutional setup?

Data security [31]:

- Does the data include PHI?
- Does the access need to be restricted?
- What is the minimum level of data security required?
- How will they be secured?
- Will the data need to be deleted after a certain time period or event? Why, when, and how?
- Are there any institutional policies or approvals to be considered?
- Who will supervise data security?
- How often (e.g., half-yearly, annually) will the adopted policies and implementations need to be reviewed and verified?

Answers to the above questions will facilitate discussions regarding optimal data storage options(s) and dictate if other institutional entities such as IT or the office of sponsored research need to be involved. Further, costs associated with data storage and reanalysis needs should also be considered when selecting storage locations. Commonly used data storage locations are personal computer(s), High Performance Computing (HPC) environments, and commercial cloud storage services (e.g., Dropbox, Box, AWS). Depending on the data, it may need to be split based on type and stored in a data-type-specific location best suited for its purpose. For example, large data such as FASTQ and VCF files may need to be stored at a location where they are accessible from an HPC environment for downstream processing, whereas spreadsheets and text documents may need to be stored in a cloud storage service (e.g., Box) to facilitate accessibility for non-computational team members.

Computing or hardware requirements will depend on the type of data and the processing planned [32]. A personal computer might suffice to process small data sets, but access to HPC or cloud computing (e.g., AWS, Microsoft Azure, Google Cloud) is often needed to process large data sets. HPCs and cloud computing offer several advantages such as fast processors, multicore chips, higher memory resources, and graphics processing units (GPU), which enable parallelization and scalability of a large number of jobs.

### Rule 6: Know what you are downloading

As outlined in Rule 2, downstream analysis can be affected by how the data was collected and processed. This becomes an even greater problem when trying to integrate multiple public data sets, each collected and processed separately. So, be mindful of the processing differences between them. In recent years, a number of projects have been established that use and share common data processing pipelines across data collections, allowing researchers to more easily combine data for more complex analyses. For example, recount3 [33] is a uniformly processed resource of hundreds of thousands of human and mouse RNA-Seq data sets designed to facilitate meta-analysis and cross-study comparisons. Another great resource is the Bgee [6] database that contains “normal,” healthy, wild-type expression data across 52 different species thereby providing a comparable reference of gene expression by anatomical entities within and between species. A third example is BioDataome [34]. BioDataome has approximately 5,600 human and mouse expression and methylation data sets pre-processed in an analogous manner to allow for direct comparisons between data. All of these data repositories provide R packages facilitating programmatic data download (see Rule 7). Collections of equivalently processed data are becoming more of the norm, but data sets of interest may not be included in them. When this is the case, it may be necessary to reprocess the data to minimize the downstream impact of differences in upstream processing.

### Rule 7: Download data programmatically; verify data integrity

Data downloads should be performed in a programmatic manner for consistency, scalability, and reliability. Several tools have been developed to provide direct programmatic access to large public databases (e.g., SRA [20], GEO [2], ENA [35], TCGA [4]) and they should be implemented when possible. One example of these tools is the SRA toolkit [36] that contains a number of commands linked to data downloads (e.g., “fasterq-dump” may be implemented to download FASTQ files and “vdb-validate” to check the integrity of SRA data sets). Additional examples include database-specific computational packages/libraries such as Bioconductor packages “recount3” [33] and “GenomicDataCommons” [37]. A key step in the data download process is validating the checksum (typically the MD5 hash) provided in the hosting database to verify data integrity prior to data analysis and interpretation. While these checks can be performed manually, some tools such as the Genomic Data Commons (GDC) Data Transfer Tool Client [37], will automatically validate MD5 checksums as part of the download process.

Depending on the number of files and tool functionality and design, customized scripts or workflows (e.g., Snakemake [38], NextFlow [39]) may be needed for scalability. When possible, publicly available workflows that adopt workflow management systems are recommended. One example is “fetchngs” [40] provided by nf-core [41]. This Nextflow pipeline [39] uses tools such as SRA-tools [36] to download FASTQ files and metadata based on a user-provided accession ID list. When performing downloads in this way, documenting the sample identifier and database sources becomes a critical step for reproducibility. For studies deposited in databases such as GEO [2], recording the GEO accession number along with sample identifiers may be sufficient; however, other data sources are updated on a continuous basis (e.g., Genotype-Tissue Expression (GTEx) Portal [5]). In such cases, the database version (e.g., GTEx Analysis Release V8—dbGaP Accession phs000424.v8.p2) and the date the download is performed should be recorded and reported in publications.

These practices should also be applied to data types beyond sample data including, for example, genome references (e.g., genome/transcriptome FASTA files, annotations files). Checksums from genomic reference files should be validated and details linked to the files should be recorded, including the FASTA file type (e.g., primary assembly or entire genomic sequence that includes assembly patches and haplotypes), database name, assembly name, and version or release number. The same principles should be applied to other reference files such as GTF/GFF [42].

### Rule 8: Do properly cite data

As with any journal article/publication information and resources used, any data generated by other researchers should be credited and properly cited. This practice benefits the original data generator by providing a tangible demonstration of value and impact beyond the initial data publication. Failing to cite the data source withholds credit from researchers in the same way that failing to cite a journal article does. Public data citations support better quality and more transparent science, making a compelling argument for other researchers to contribute their own data to public data repositories. This process also supports improved reproducibility and credibility for your own research.

When it comes to citing data, the field still lacks a gold standard. However, existing best practices include citing the original paper where the data was published or a data object identifier (DOI) generated from a persistent database like Zenodo [43] or figshare [44]. In addition, consortium projects such as GTEx [5] and TCGA [4] often provide guidance on citation practices for their repositories. In an effort to increase the citability of public data and establish the data itself as a scientific output of value separate from the associated manuscript, many researchers now publish data sets independently from their associated publications. Journals for this purpose, such as *Scientific Data*, now exist [45]. If there is no guideline associated with the data set in general the citation should include the generators, where the data was obtained from, accession numbers, the version number for the data (if applicable), and the date it was accessed.

Non-profit organizations such as DataCite and Crossref provide unique persistent identifiers (PIDs) to data sets to improve tracking usage and facilitate linking to the publication of origin [46]. Check and see if the public data you are using in your analysis has a PID that can be cited or included in the methods section. In the future, PIDs may perhaps be linked to an individual’s ORCID number to provide a standardized data citation approach. A study looking at the correlation between if data was publicly available and the citation rate of the original paper demonstrated that those including publicly available data within the paper was associated with a 69% increase in citations [47]. Furthermore, investigators who share public data sets well have an increase in the impact of their own research. Articles with links to data repositories or that include PIDs are more highly cited than those without [24]. In summary, public data use benefits both the creator and the user.

### Rule 9: Make your reprocessed data and models FAIR and share

All of the rules mentioned above are possible to adhere to because researchers made their data Findable, Accessible, Interoperable, and Reusable (FAIR) [48]. As a contributing member of the research ecosystem, you should pass it on, too! Make sure any additional data you generate, reuse, and integrate (i.e., data models), adheres to the FAIR principles. recount3 [33] and Bgee [6] are good examples of public data that have been reprocessed to correct for batch effects and made available under FAIR data principles. If your research is funded through the NIH, then you must adhere to a Data Management and Sharing Plan as outlined in the NIH policy starting in 2023 [10]. Recent research shows that only 6.8% of authors respond to requests for data sharing dramatically reducing the impact of most data and the knowledge to be gained from its use [49]. To be FAIR:

- Improve data findability: As highlighted in Rule 8, submit your data to stable open-access public data repositories that provide DOIs such as Zenodo [43] and figshare [44]. Personal, lab, group, or institute sites are not good long-term solutions. Socialize your data by sharing on social media platforms such as Twitter. Blogs and news articles on lab and/or institute sites and presenting at conferences where data are clearly identified with DOIs help others to discover useful data. By diversifying where and how you share with the community about your data, you cast the widest net in order to catch the attention of potential researchers who would also benefit from access to your data.
- Improve data accessibility: Open access to publications is important to science and plays a critical role in reproducing and advancing science. Making your data readily accessible is equally important. Share your raw as well as processed data so that the analysis performed can be reproduced fully and with minimal effort.
- Improve data interoperability: Interoperability refers to the ability of data from different sources to be able to integrate with minimal effort [48]. A good example is the Fast Health Interoperability Resources (FHIR) standard for health care data exchange [50]. This becomes even more critical in studies where data from different sources are being reused, so make sure the data you provide is highly interoperable by including relevant metadata and adhering to appropriate and reasonable file and data conventions within the field.
- Improve data reuse: Make your data reuse, reusable. Providing data in standard and popular formats goes a long way in making it reusable. Incomplete metadata and methods can severely limit the reuse (and usefulness) of data. Don’t skimp by providing the bare minimum data and metadata needed to satisfy the requirements of the granting body, governing body, journal, etc., that you’re looking to communicate your work through. Abstaining from providing all necessary assets to reproduce the work does a disservice to you, your colleagues, your lab and institute, and the scientific community. It is not just about the input and the output: There’s a whole bunch of research, development, refinement, knowledge, and other work done in between data input and output that needs to be captured, codified, and shared with the work itself.

### Rule 10: Make your processing, pipelines, and code FAIR

Tools and methods used for the analysis and interpretation of public data sets should also adhere to the FAIR guidelines for coding and software development [51]. Many of the FAIR principles for data (outlined in Rule 9) are directly applicable to software, but others require modification for application to software [52]. For example, persistent identifiers should be generated and recorded for novel pipelines, software, and research tools. Findability of software should be linked to the traceability of the source code under version control (e.g., GitHub [53], GitLab [54], BitBucket [55]) and reporting of appropriate metadata such as software versions. Similarly, software and code associated with an analysis should be accessible through repositories and (when appropriate) software archives such as CRAN [56], Bioconductor [57], PyPI [58], and Conda [59]. The same is true for software dependencies. Software containers (e.g., Docker [60], Singularity [61]) can also be implemented to enable software portability and analysis reproducibility. In the absence of containerized software, the necessary information for how to build and install a published tool should be provided.

Because the dynamic nature of software can mean the most up to date version is different from the version that was published, proper documentation and tagging (e.g., GitHub tags linked to released versions of software) allows researchers to find the exact package versions used during a study, therefore facilitating reproducible analyses. Continuous integration approaches (the software development practice of automating builds, static analysis, and tests on code changes that were pushed to a central repository) can automate time-consuming tasks associated with pipeline and software development. Similarly, static code analysis tools in continuous integration pipelines can help automate source code quality analysis allowing early identification of potential bugs, security vulnerabilities, performance issues, or deviations from the project/organization and coding guidelines.

Inclusion of continuous integration and static code analysis tools allow for rapid feedback loops in code inspection, reducing the time reviewers need to spend reviewing, and reducing cost of time and funding of development maintenance [62,63]. Journals are increasingly requiring that the code used for analysis is made available [64]. In addition to ensuring that your processing, pipelines, and code are FAIR, the steps above will help to ensure code review is not an onerous task being performed during the submission process and your methods are reproducible for other scientists [65–67]. For more detailed information about how you can make your research more computationally reproducible, refer to [68].

## Conclusion

In summary, biological data reuse is not only good for science, but also it is the right thing to do in order to extract the greatest societal impact from the samples and funding that patients, donors, and taxpayers generously provide. Here, we covered Ten Simple Rules for data reuse spanning the periods before, during, and after data download. This paper serves as a guide for both data users and generators in the community.
